# Identifying the Speech Production Stages in Early and Late Adulthood by Using Electroencephalography

**DOI:** 10.3389/fnhum.2019.00298

**Published:** 2019-09-10

**Authors:** Jakolien den Hollander, Roel Jonkers, Peter Mariën, Roelien Bastiaanse

**Affiliations:** ^1^International Doctorate in Experimental Approaches to Language and Brain (IDEALAB, Universities of Groningen, Potsdam, Newcastle, Trento and Macquarie University), Sydney, NSW, Australia; ^2^Center for Language and Cognition Groningen (CLCG), University of Groningen, Groningen, Netherlands; ^3^Clinical and Experimental Neurolinguistics (CLIEN), Vrije Universiteit Brussel, Brussels, Belgium; ^4^Department of Neurology and Memory Clinic, ZNA Middelheim General Hospital, Antwerp, Belgium; ^5^Center for Language and Brain, National Research University Higher School of Economics, Moscow, Russia

**Keywords:** speech production, aging, electroencephalography, word retrieval, articulation

## Abstract

Structural changes in the brain take place throughout one’s life. Changes related to cognitive decline may delay the stages of the speech production process in the aging brain. For example, semantic memory decline and poor inhibition may delay the retrieval of a concept from the mental lexicon. Electroencephalography (EEG) is a valuable method for identifying the timing of speech production stages. So far, studies using EEG mainly focused on a particular speech production stage in a particular group of subjects. Differences between subject groups and between methodologies have complicated identifying time windows of the speech production stages. For the current study, the speech production stages lemma retrieval, lexeme retrieval, phonological encoding, and phonetic encoding were tracked using a 64-channel EEG in 20 younger adults and 20 older adults. Picture-naming tasks were used to identify lemma retrieval, using semantic interference through previously named pictures from the same semantic category, and lexeme retrieval, using words with varying age of acquisition. Non-word reading was used to target phonological encoding (using non-words with a variable number of phonemes) and phonetic encoding (using non-words that differed in spoken syllable frequency). Stimulus-locked and response-locked cluster-based permutation analyses were used to identify the timing of these stages in the full time course of speech production from stimulus presentation until 100 ms before response onset in both subject groups. It was found that the timing of each speech production stage could be identified. Even though older adults showed longer response times for every task, only the timing of the lexeme retrieval stage was later for the older adults compared to the younger adults, while no such delay was found for the timing of the other stages. The results of a second cluster-based permutation analysis indicated that clusters that were observed in the timing of the stages for one group were absent in the other subject group, which was mainly the case in stimulus-locked time windows. A *z*-score mapping analysis was used to compare the scalp distributions related to the stages between the older and younger adults. No differences between both groups were observed with respect to scalp distributions, suggesting that the same groups of neurons are involved in the four stages, regardless of the adults’ age, even though the timing of the individual stages is different in both groups.

## Introduction

### Effects of Aging on the Brain

Structural changes in the brain, such as a reduction in cortical thickness (Freeman et al., 2008; Zheng et al., 2018), a decrease in the number of cortical folds (Zheng et al., 2018), and a reduction in gray (Freeman et al., 2008) and white matter (Marner et al., 2003) take place throughout one’s lifetime. Also, the connectivity within the cingulo-opercular network [CON; including dorsal anterior cingulate, medial superior frontal cortex, anterior insula, frontal operculum, and anterior prefrontal cortex (Dosenbach et al., 2007)] and the frontoparietal control network [FPCN; including the lateral prefrontal cortex, anterior cingulate cortex, and inferior parietal lobule (Vincent et al., 2008)] reduces with aging (Geerligs et al., 2015). These networks modulate higher cognitive functions involved in language processing, such as working memory and reading. While the global efficiency of the three networks is the same in older and younger adults, the local efficiency and the modularity decrease with aging. This decrease may delay the speech production process; however, the efficiency of the visual network, which is used when watching pictures, is maintained. Therefore, no delay in the processing of information has been observed in the visual network with aging.

Age-related changes in the brain are also reflected in the oscillations of the brain, which can be measured using electroencephalography (EEG). The amplitude of components (peaks that are related to a particular process in the brain) in the processed signal, observed when many neurons fire together, is reduced in older individuals (Wlotko et al., 2010). There are two reasons why this reduction may occur: (1) neurons that fire together are geometrically less aligned and do no longer fire synchronously and (2) the latency of the component is more variable. Also, delays in the latency of the N400 component have been observed in older individuals. According to the global slowing hypothesis (Brinley, 1965), older adults are slower in every process, which should be reflected in the EEG. Slower processing speed may, thus, be observed in older adults when carrying out a cognitive task, because they cannot focus on speed when they are focusing on responding as accurately as possible, known as the “speed–accuracy tradeoff” (Ratcliff et al., 2007). Not being able to focus on both speed and accuracy is possibly related to a decrease in the strength of the tract between the presupplementary motor area and the striatum in older adults (Forstmann et al., 2011).

### Effects of Aging on the Speech Production Process

Between 25 and 100% of the structural and functional changes in the brain are related to cognitive decline (Fjell and Walhovd, 2011). Cognitive decline caused by aging may have an effect on the speech production process. For example, older adults are less accurate in picture naming than younger adults (Connor et al., 2004). Decline in object naming is accompanied by a reduction in white and gray matter in the left temporal lobe (Cardenas et al., 2011). The temporal lobe has been associated with semantic memory, in which concepts are stored. When a concept activates a lemma (the word meaning) in the lexicon, semantically related lemmas get coactivated. The correct lemma is retrieved from the mental lexicon when lemmas that are semantically related to the target are sufficiently inhibited. Both semantic memory and inhibition decline with aging (Harada et al., 2013).

After the lemma retrieval stage, the lexical word form, the lexeme, is retrieved. When there is insufficient information available about the lexeme, the phonological form of the word cannot be retrieved. The speaker experiences a temporal failure to produce a word even though the word is well known to him. This so-called tip-of-the-tongue phenomenon is observed more frequently in older adults, particularly in those with atrophy in the left insula (Shafto et al., 2007).

In the next stage of object naming, phonological encoding, the phonemes corresponding to the lexeme are retrieved and ordered and the phonological rules are applied. No aging effects have been reported for phonological encoding. Finally, the string of phonemes is phonetically encoded into an articulation plan. This plan specifies how the muscles of the mouth and throat will interact during the articulation of the word. Older individuals have a longer response duration for the production of both sequential and alternating syllable strings, which is associated with reduced cortical thickness in the right dorsal anterior insula and in the left superior temporal sulcus and gyrus (Tremblay and Deschamps, 2016).

**FIGURE 1:**
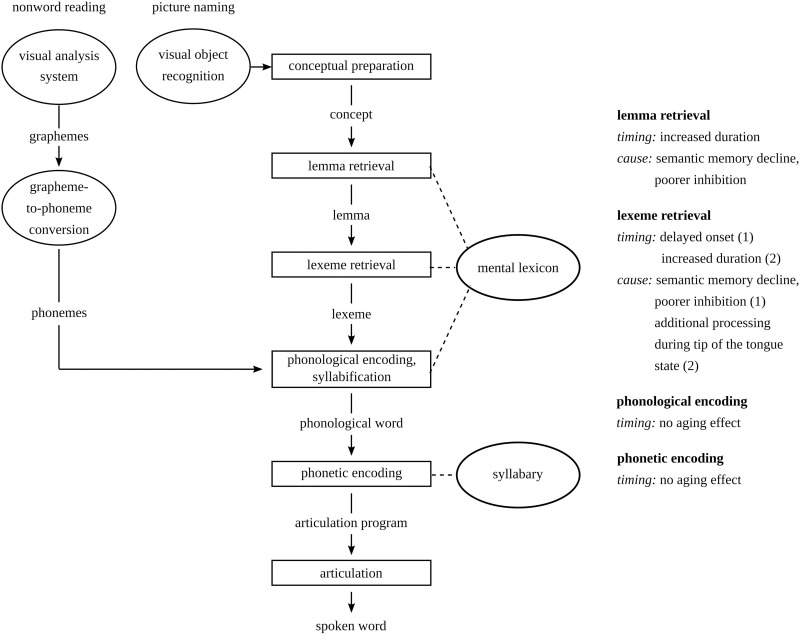
Stages in the model of spoken word and non-word production based on Levelt et al. (1999) and how they may change in later compared to earlier adulthood.

In sum, delayed lemma retrieval can be observed in older individuals (Cardenas et al., 2011) due to reduced semantic memory and poorer inhibition abilities (Harada et al., 2013). A delay at the lemma level may delay the onset of lexeme retrieval. Lexeme retrieval may be delayed due to tip-of-the-tongue states (Shafto et al., 2007). In this study, lemma and lexeme retrieval are studied in picture-naming tasks, while phonological and phonetic encoding are studied in non-word production tasks. Since lemma and lexeme retrieval do not play a role in non-word production tasks, delays in these stages cannot delay the onset of phonological and phonetic encoding. Aging is not expected to have an effect on these two stages, because no aging effects on phonological encoding have been reported. Also, the task used to study phonetic encoding is different from the task used by Tremblay and Deschamps (2016). An overview of the stages in spoken word and non-word production that may change in later adulthood is provided in Figure 1.

### Current Study

The hypothesis that the lemma and lexeme retrieval stages are delayed in older compared to younger individuals, whereas phonological and phonetic encoding are similar in both groups, can be tested using EEG. Since each speech production stage has its own timing (Indefrey, 2011), it is possible to identify the individual stages using tasks in which more processing is required at the particular stage. Lemma retrieval requires more effort when the number of previously retrieved lemmas from neighboring nodes increases. This effect is referred to as the “cumulative semantic interference effect” (Howard et al., 2006). Two EEG studies have used this effect to target the stage of lemma retrieval, which has been identified from 150 to 225 ms (Maess et al., 2002) and from 200 to 380 ms after stimulus presentation (Costa et al., 2009).

Lexeme retrieval requires more effort when the age of acquisition (AoA) of words increases (Laganaro and Perret, 2011; Laganaro et al., 2012; Valente et al., 2014). This stage has been identified in a time window from 120 to 350 ms after stimulus presentation and around 280 and 150 ms before response onset (Laganaro and Perret, 2011), from 380 to 400 ms after stimulus presentation and up to 200 ms before response onset (Laganaro et al., 2012), and from 380 after stimulus presentation up to 100 ms before response onset (Valente et al., 2014).

Phonological encoding requires more effort when the number of phonemes increases. So far, word length effects have not been identified in EEG studies, meaning that the time frame of phonological encoding has not been identified yet using this manipulation (Valente et al., 2014; Hendrix et al., 2017). However, other tasks, such as comparing overt and covert production of nouns and verbs, have been used to track phonological encoding (Sahin et al., 2009). In the current study, non-word length is used, which may lead to different findings.

Syllable frequency is known to have an effect on phonetic encoding: when syllable frequency decreases, phonetic encoding requires more effort (Levelt and Wheeldon, 1994). In a task in which phonemes were inserted into non-words with varying frequencies in a non-word reading task, the syllable frequency effect has been identified using EEG from 170 to 100 ms before response onset (Bürki et al., 2015). Our methodology is different because participants were asked to read the non-words, not to insert phonemes. It is, therefore, unclear what to expect.

Hence, for the current study, the cumulative semantic interference effect, the AoA effect, the effect of non-word length in phonemes, and the syllable frequency effect will be used to track the speech production stages in a group of younger adults and in a group of older adults. The time windows of the stages in both groups will be identified. If the time windows of the stages differ between the two groups, that does not mean that the processing mechanisms are different (Nieuwenhuis et al., 2011). Therefore, a direct comparison of both groups will be made in the time windows of the relevant stages that were identified in the younger adults and the older adults. Additionally, the scalp distributions of the stages will be compared between the two groups.

## Materials and Methods

### Participants

For the group of young adults, 20 young adulthood native speakers of Dutch (5 males) participated. The mean age of the participants was 21.8 years (age range: 17–28 years). Participants in the group of older adults were 20 late adulthood native speakers of Dutch (7 males). Their average age was 55.4 years (range: 40–65). The young adult participants are referred to as “younger adults,” and the late adulthood participants are referred to as “older adults.” The younger adults’ data will be the basis of this study, and their data will be compared to those of the older adults.

All participants were right handed, measured using the short version of the Edinburgh Handedness Inventory (Oldfield, 1971). They reported no problems in hearing, and their vision was normal or corrected to normal. Also, they reported no reading difficulties. All participants were financially compensated and gave informed consent. The study was approved by the *Ethics Committee of Humanities* of the University of Groningen.

### Materials

#### Lemma Retrieval

The materials used in the lemma retrieval task were black-and-white drawings. The pictures originated from the *Auditief Taalbegripsprogramma* (*ATP*; Bastiaanse, 2010) and the *Verb and Action Test* (*VAT*; see Bastiaanse et al., 2016) for individuals with aphasia. The order in which the depicted nouns were presented was manipulated for the cumulative semantic interference effect. The pictures were grouped in sets of five semantically related neighbors (e.g., bed, couch, cradle, closet, and chair) that fit into a particular category (e.g., furniture, clothes, and insects). The five nouns within one category had the same number of syllables and the same stress pattern and were controlled for logarithmic lemma frequency in Dutch (Baayen et al., 1995). The depicted nouns were all mono- or disyllabic in Dutch.

For the selection of the final item list, a picture-naming task was carried out by four participants (one male) with a mean age of 22 years (age range: 21–23 years). Items that were named incorrectly by more than one participant were removed. The 125 selected items had an overall name agreement of 91.4%. The overall mean logarithmic lemma frequency was 1.28 (range: 0–2.91). The same set of pictures was used in two lists with reversed conditions to avoid an order of appearance effect. The lists were presented in three blocks of 30 items and one block of 35 items.

The pictures were presented on a computer screen, and participants were asked to name the pictures as quickly and accurately as possible. Before the picture was presented, a black fixation cross on a white background was shown for 500 ms. The function of the fixation cross was to draw attention and to announce that a picture was presented soon. The picture was shown for 5 s. Items within one category were not presented directly after another.

#### Lexeme Retrieval

The pictures for this test originated from the same sources as the materials on the first test and represented mono- and disyllabic nouns in Dutch. Items were controlled for AoA (Brysbaert et al., 2014) and lexeme frequency (Baayen et al., 1995).

Four participants (one male) with a mean age of 20.7 years (age range: 19–22) took part in a picture-naming task for pretesting the materials. These participants had not taken part in the lemma retrieval task. Items that were named incorrectly by more than one participant were omitted.

The 140 selected items had an overall name agreement of 93.9%. AoA ranged from 4.01 years for the noun “book” to 9.41 years for the noun “anchor,” with a mean of 5.96 years. The mean logarithmic lexeme frequency was 1.02 (range: 0–2.44). The correlation between AoA and lexeme frequency in the items is significant [*r*(138) = −0.28, *p* < 0.001]. Therefore, in the analysis, only AoA has been taken into account. The items were organized in one list including four blocks of 35 items. The order of the items was randomized per block, so that every participant named the items in a different order.

The procedure of the lexeme retrieval task was the same as the procedure of the lemma retrieval task. Since there was some item overlap between the lemma and lexeme retrieval tasks, the two tasks were never administered consecutively. A non-word task was always administered in between.

#### Phonological and Phonetic Encoding

To identify the stages of phonological and phonetic encoding, a non-word reading task was used.^1^ All non-words were disyllabic and composed of existing Dutch syllables. The combination of the two syllables resulted in a non-word, e.g., “kikkels” or “raalkro.” The non-words were controlled for spoken syllable frequency (Nederlandse Taalunie, 2004). Two lists of non-words were developed in written form for the reading task. The two lists contained the same syllables, but the syllables were combined differently; thus, the non-words were unique.

The non-words were pretested in a reading task by four participants who took part in pretesting the picture-naming tasks as well. Each list was pretested with two participants. The 140 selected items for list 1 had an accuracy rate of 100%; 8% of the non-words in list 2 were produced incorrectly. The syllables used in these items were combined into new non-words. These non-words were pretested again with two other participants. Their accuracy was 100%.

For each non-word, the average spoken syllable frequency was computed over its two syllables. For list 1, the mean frequency was 1,136 (range: 257–4,514) and 1,077 (range: 257–4,676) for list 2. Also, the number of phonemes in the non-words was controlled for, because the duration of phonological encoding may increase with the number of phonemes. For both lists, the number of phonemes in the non-words ranged from 3 to 8. The average number of phonemes was 5.33 for list 1 and 5.29 for list 2.

The non-words were presented in white letters on a black background. The font type Trebuchet MS Regular, size 64, was used. The stimulus was presented for 5 s and preceded by a fixation cross, which was presented for 500 ms. Participants read either list 1 or list 2. Each list was divided into four blocks of 35 items. The order in which the non-words was presented was randomized per block, so none of the participants read the non-words in the same order. The instruction was to read the non-words aloud as quickly and accurately as possible.

### General Procedure

During the experiments, participants were seated approximately 70 cm from the screen. E-Prime 2.0 (2012) was used to present the stimuli and to record the response times and the responses. A voice key was used to detect the response times. The responses were recorded using a microphone that was attached to a headset. Before the experiment started, participants practiced the task with five items for the picture-naming tasks and with eight items for the non-word reading task. Participants had the opportunity to take a short break between the four blocks of the experiments.

#### EEG Data Recording

Electroencephalography data were recorded with 128 (older adults) and 64 (younger adults) Ag/AgCl scalp electrodes (WaveGuard) cap using the EEGO and ASA-lab system (ANT Neuro Inc., Enschede, Netherlands). These systems are entirely compatible; EEGO is the latest version. For the older adults, only the 64 channels that were recorded in the younger group were analyzed. The full set of 128 electrodes was used in a different study. The electrode sites were distributed over the scalp according to the 10-10 system (Jasper, 1958) for the system with 64 electrodes and according to the 10-5 system for the system with 128 electrodes. Bipolar electrodes were used to record vertical ocular movements, such as eye blinks, for which the electrode sites were vertically aligned with the pupil and located above and below the left eye. Impedance of the skin was kept below 20 kΩ, which was checked before every experiment. Data were acquired with a sampling rate of 512 Hz, and reference was recorded from the mastoids.

### Data Processing and Analysis

#### Behavioral Data

The audio recordings of the participants’ responses were used to determine the speech onset time. The speech onset time in each audio file was manually determined using the waveform and the spectrogram in Praat (Boersma and Weenink, 2018). The speech onset times based on the audio files were used as response events in the response-locked EEG analysis. R was used for the statistical analysis of the behavioral and item data (R Core Team, 2017).

Trials to which participants responded incorrectly were excluded from the analysis (lemma retrieval: 7.8%; lexeme retrieval: 7.3%; phonological and phonetic encoding: 1.9%). Also, responses that included hesitations or self-corrections qualified as errors (lemma retrieval: 2.6%; lexeme retrieval: 2.6%; phonological and phonetic encoding: 0.8%). Items to which many participants responded extraordinarily fast or slow were excluded from the EEG analysis (lemma retrieval: 8%; lexeme retrieval: 18.6%; phonological and phonetic encoding: 12.1%). The average response time was computed over all accepted trials. Trials exceeding this average by 1.4 standard deviations were disregarded.

#### EEG Data

The EEG data were preprocessed using EEGLAB (Delorme and Makeig, 2004) as an extension to MATLAB (2015). After rereferencing to the average reference of the mastoids, the data were filtered with a 50-Hz notch filter to remove electricity noise and bandpass filtered from 0.2 to 30 Hz. Then, the data were resampled to 128 Hz. Independent components analysis on all channels was used for artifact detection. Artifact components, such as eye blinks, were removed through visual inspection. Also, the effect of component removal on the data was visually inspected. The continuous data were segmented per trial from 200 ms until 2 s after stimulus onset. A baseline correction was applied over the data epochs, using the 200 ms before stimulus onset as a baseline. Then, the events of disregarded trials were removed. To study the time window from the stimulus onset until the response onset, both stimulus-locked analyses, in which the time window after stimulus onset is analyzed, and response-locked analyses, in which the backward time window before the response onset is analyzed, were carried out. For the stimulus-locked analysis, the data epochs were segmented from stimulus onset until one sampling point (8 ms) after the earliest response time. This one extra sampling point was removed before the analysis. The start of the response-locked analysis was determined by subtracting the stimulus-locked time window from the response onset. Depending on the task, accepted trials were coded into two or three conditions for the statistical analysis. The conditions are specified below per experiment. These data were exported from EEGLAB into the format used in FieldTrip (Oostenveld et al., 2011), which was used for the statistical analysis. Finally, the structure of the data files was prepared for a cluster-based permutation analysis (Maris and Oostenveld, 2007).

The aims of the analyses were to identify the time window of lemma retrieval with the cumulative semantic interference effect, the time window of lexeme retrieval with the AoA effect, the time window of phonological encoding with the non-word length in phonemes effect, and the time window of phonetic encoding with the syllable frequency effect. These time windows were identified in the group of older adults and in the group of younger adults using group-level cluster-based permutation analyses carried out over all participants per group. The cumulative semantic interference effect was computed as the difference between the first and the fifth presented item within a category. The difference between words with an AoA of around 5 years and words with an AoA of around 6 years, as well as the difference between words with an AoA of 5 years and words with an AoA of around 7 years were used to compute the AoA effect. The effect of non-word length in phonemes was computed as the difference between non-words consisting of four phonemes and non-words consisting of five phonemes, as well as the difference between non-words consisting of four phonemes and non-words consisting of six phonemes. The difference between non-words with a high syllable frequency of 1,000–1,500 and non-words with a moderate syllable frequency of 500–1,000, as well as the difference between non-words with a high syllable frequency of 1,000–1,500 and non-words with a low syllable frequency of 250–500 were used to compute the syllable frequency effect. In every analysis, the number of permutations computed was 5,000. The Monte Carlo method was used to compute significance probability, using a two-sided dependent samples *t*-test (α = 0.025). In the first analysis of every experiment, the entire time window from stimulus onset until 100 ms before response onset was tested. When an effect was revealed in this large time window, a smaller time window around the effect was tested once, so a more specific timing of the effect could be reported. Finally, the time windows of the stages in older and younger adults were compared. This method cannot show whether the two groups differ (Nieuwenhuis et al., 2011). Therefore, the EEGs of both groups have been compared in the time windows of the stages for every single condition using a cluster-based permutation analysis. Again, the Monte Carlo method was used to compute significance probability, but now a two-sided independent samples *t*-test (α = 0.025) was used to compare the two subject groups.

Additionally, a *z*-score mapping analysis (Thatcher et al., 2002) was carried out to compare the scalp distributions of the older adults to those of the younger adults during the speech production stages. For each experiment, the data were analyzed in relevant time windows and conditions for which significant clusters were found in the cluster-based permutation analysis of the older and the younger adults. The length of these time windows varied between the participant groups, which would have caused a difference in the number of time points included in the analysis. To avoid this difference, the number of time points centered around the median of the longest time window used in the analysis was made equal to the number of time points in the shortest time window. For each time point, *z*-scores were computed per electrode. The mean computed over the younger adults’ data was subtracted from each data point from the older adults’ data individually. This subtraction was divided by the standard deviation computed over the younger adults’ data. Mean *z*-scores were computed per condition. When the mean *z*-score deviated more than one standard deviation from zero, the difference between the age groups qualified as significant.

## Results

The mean, standard deviation, and range of the response time data from the three experiments are provided per participant group in Table 1. For all analyses on response time, only the correct responses were used.

**TABLE 1:** Response times of the younger and older adults.

**Task**	**Mean (ms)**		**Standard deviation (ms)**		**Range (ms)**	
			
	**young**	**old**	**young**	**old**	**young**	**old**
Lemma retrieval	932	944	216	213	602–1461	603–1460
Lexeme retrieval	938	946	199	201	626–1440	628–1439
Phonological and phonetic encoding in reading	690	699	116	119	502–966	504–965

### Behavioral Results

#### Younger Adults

At all tasks, the younger adults performed at ceiling. The percentages of correct responses were 92.4% for lemma retrieval, 92.9% for lexeme retrieval, and 98% for the non-word reading task targeting phonological and phonetic encoding. On the lemma retrieval task, a cumulative semantic interference effect was found on the response time [*F*(1, 765) = 13.38, *p* < 0.001]. Increased response times were found for pictures within a category that were presented at the fifth ordinal position compared to pictures that were presented at the first ordinal position. An AoA effect on the response time was identified on the lexeme retrieval task [*F*(1, 2,205) = 104.01, *p* < 0.001]. Response time increased as AoA advanced. Non-word length in number of phonemes is relevant at the level of phonological encoding and turned out to be a significant factor: response times increased when non-words consisted of more phonemes [*F*(1, 2,096) = 5.71, *p* = 0.017]. The frequency of the syllables was varied to tap into phonetic encoding. Response times were found to decrease when syllable frequency increased [*F*(1, 2,320) = 6.35, *p* = 0.01].

#### Older Adults

Like the younger adults, the older adults performed at ceiling on all tasks. The percentages of correct responses were 86.8% for lemma retrieval, 87.6% for lexeme retrieval, and 96.5% for the non-word reading tasks. A cumulative semantic interference effect was found on the lemma retrieval task [*F*(1, 721) = 7.60, *p* = 0.006]. Increased response times were found for pictures within a category that were presented at the fifth ordinal position compared to those presented at the first ordinal position. Also, increased response times were found for items with a later AoA on the task targeting lexeme retrieval [*F*(1, 2,061) = 43.38, *p* < 0.001]. In the non-word reading task, response times increased with the non-word length in number of phonemes, which was used as a marker for *phonological encoding* [*F*(1, 1,943) = 5.60, *p* = 0.018]. Furthermore, to target phonetic encoding, a decrease in syllable frequency of the non-words was found to increase response times [*F*(1, 2,146) = 11.68, *p* < 0.001].

#### Differences Between Younger and Older Adults

On *all* tasks, differences in response times between both age groups were found. The older adults responded slower than the younger adults on the lemma retrieval task [*F*(1, 1,488) = 4.81, *p* = 0.028], the lexeme retrieval task [*F*(1, 4,268) = 7.14, *p* = 0.007], and the non-word reading task targeting phonological and phonetic encoding [*F*(1, 4,468) = 28.58, *p* < 0.001]. Moreover, an interaction effect of AoA and participant age was found [*F*(1, 4,268) = 4.51, *p* = 0.034]. The group of older adults showed a smaller AoA effect [*F*(1, 2,061) = 43.38, *p* < 0.001] than the group of younger adults [*F*(1, 2,205) = 104.01, *p* < 0.001].

### EEG Results

For the presentation of the EEG results, we will first present the results of the cluster-based permutation analysis for each task in the younger adults and then in the older adults to identify the time windows of the effects in these groups. Then, the differences between the two groups in these time windows computed with cluster-based permutation analyses will be presented along with the comparisons of the scalp distributions of both age groups. The EEG statistics are given in Appendix 1A (younger adults), Appendix 1B (older adults), and Appendix 1C (comparison of older and younger adults).

#### Younger Adults

**FIGURE 2:**
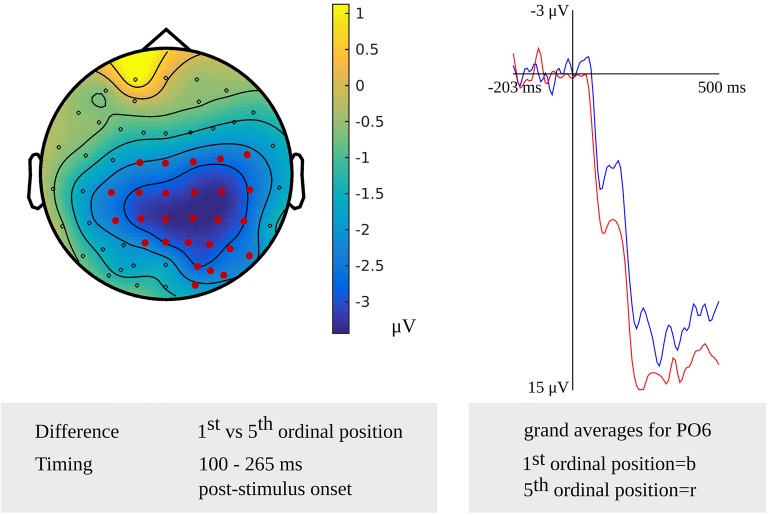
**Left**: The cluster related to the cumulative semantic interference effect in the younger adults that was revealed in the stimulus-locked analysis of the lemma retrieval task. Electrodes included in the cluster are marked in red. **Right**: The waveforms of the grand averages for the 1st (in blue) and 5th ordinal position (in red) for electrode PO6 in the younger adults.

In the younger adults, a difference between the first and fifth ordinal positions that was taken as evidence for the stage of lemma retrieval was revealed in the latency range from 100 to 265 ms (*p* = 0.005) after stimulus onset. The difference was most pronounced over right central and posterior sensors. In the response-locked analysis, an effect was found from 445 to 195 ms (*p* = 0.004) before response onset. The effect was most pronounced over central and posterior sensors bilaterally and over the right frontal electrodes. The scalp distribution of the stimulus-locked effect and the waveforms of the grand averages for the first and fifth ordinal position are shown in Figure 2.

**FIGURE 3:**
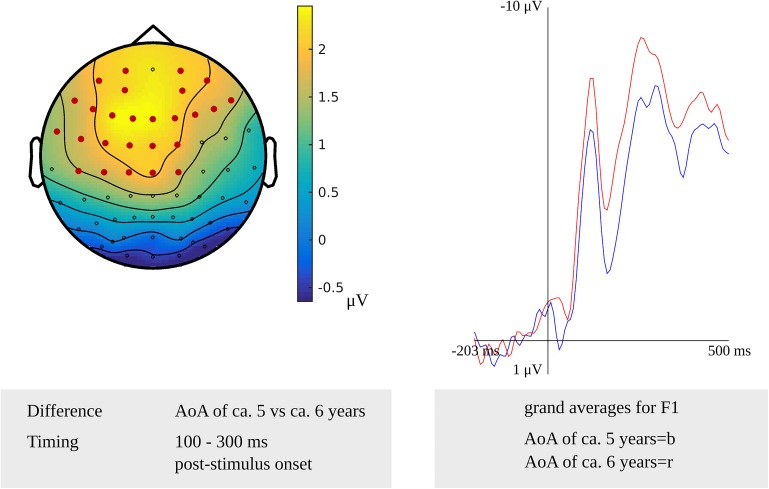
**Left**: The cluster related to the AoA effect in the younger adults that was revealed in the stimulus-locked analysis of the lexeme retrieval task. Electrodes included in the cluster are marked in red. **Right**: Waveforms of the grand averages for an AoA of ca. 5 (in blue) and 6 years (in red) for electrode F1 in the younger adults.

Testing for an AoA effect targeting lexeme retrieval in the latency range from 100 to 300 ms after stimulus onset in the younger adults, the cluster-based permutation test revealed a difference between the items with an early AoA and items with a moderate AoA (*p* = 0.002). The difference was most pronounced on bilateral frontal and central sensors, as shown in Figure 3. Figure 3 also shows the waveforms of the grand averages for the early and moderate AoA conditions. In the response-locked cluster-based permutation analysis, a difference between items with an early AoA and items with a late AoA was revealed from 475 to 330 ms before response onset. The response-locked AoA effect was most pronounced on bilateral frontal and bilateral central electrodes (*p* < 0.001).

**FIGURE 4:**
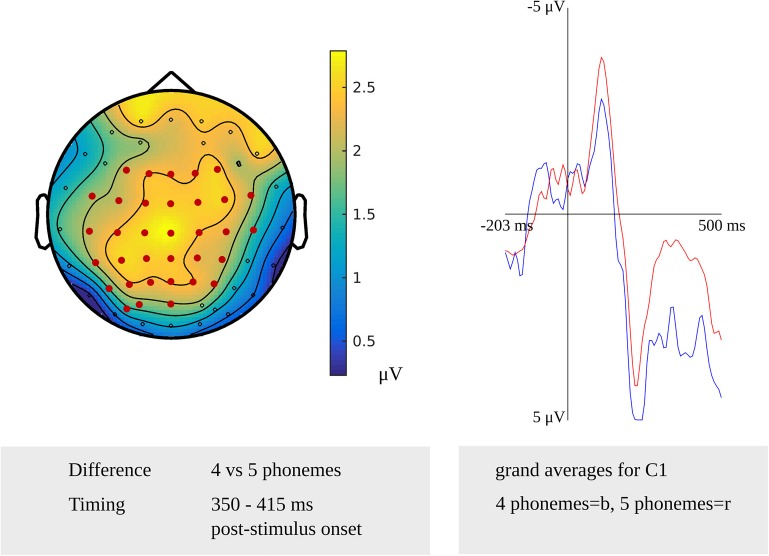
**Left**: The cluster related to the effect of non-word length in the younger adults that was revealed in the stimulus-locked analysis of the task targeting phonological encoding. Electrodes included in the cluster are marked in red. **Right**: Waveforms of the grand averages for a non-word length of four (in blue) and five phonemes (in red) for electrode C1 in the younger adults.

A stimulus-locked length effect was revealed from 350 to 415 ms for the comparison of non-words consisting of four and five phonemes (*p* = 0.0032) targeting phonological encoding, which is shown in Figure 4. The waveforms of the grand averages for non-word length in four and five phonemes are provided in Figure 4 as well. Also, a stimulus-locked length effect was revealed as a difference between non-words consisting of four and six phonemes in a time window from 390 to 425 ms after stimulus presentation (*p* = 0.0046). Both stimulus-locked effects were most pronounced over the bilateral centro-posterior electrodes. In the response-locked analysis, a length effect was identified as a difference between four and five phonemes from 335 to 320 ms before response onset, which was most pronounced over bilateral central and left posterior electrodes (*p* = 0.0084). Also, a length effect for the difference between four and six phonemes was revealed from 330 to 320 ms before response onset (*p* = 0.0084). This effect was most pronounced in right central and bilateral posterior electrodes.

**FIGURE 5:**
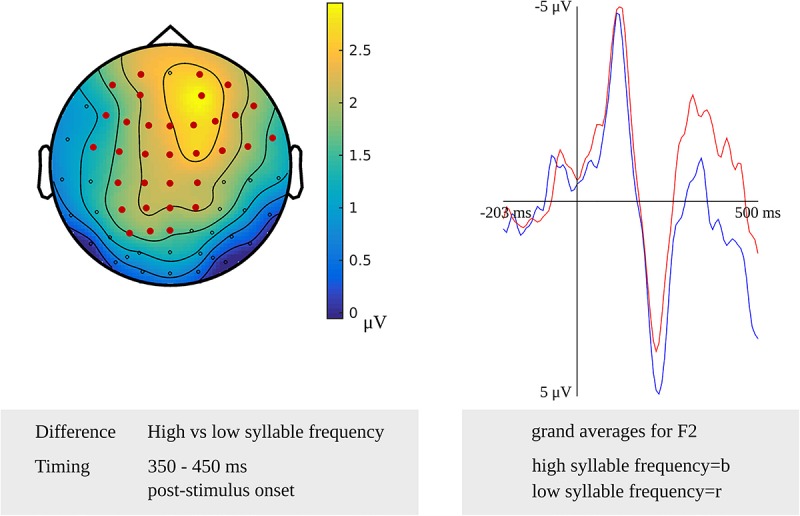
**Left**: The cluster related to the syllable frequency effect in the younger adults that was revealed in the stimulus-locked analysis of the task targeting phonetic encoding. Electrodes included in the cluster are marked in red. **Right**: Waveforms of the grand averages for high (in blue) and low syllable frequency (in red) for electrode F2 in the younger adults.

Testing for a syllable frequency effect targeting phonetic encoding in the latency range from 400 to 450 ms after stimulus onset in the younger adults, the cluster-based permutation test revealed a difference between items with a high syllable frequency and items with a moderate syllable frequency (*p* = 0.020). In this latency range, the difference was most pronounced over the central sensors bilaterally. Another stimulus-locked syllable frequency effect was found as a difference between items with a high syllable frequency and items with a low syllable frequency in a time window from 350 to 450 ms after stimulus onset (*p* = 0.012), which is shown in Figure 5. The difference was most pronounced at the frontal and central sensors bilaterally. In Figure 5, the waveforms of the grand averages for the high and low syllable frequency items are provided as well. In the response-locked analysis, a difference between items with a high syllable frequency and items with a low syllable frequency was revealed in a time window from 250 to 200 ms before response onset (*p* = 0.021). The effect was most pronounced at bilateral central sensors.

#### Older Adults

**FIGURE 6:**
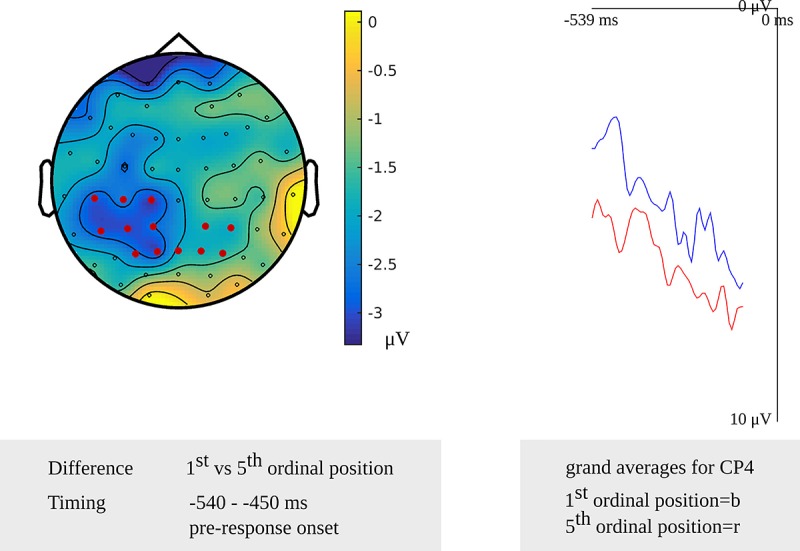
**Left**: The cluster related to the cumulative semantic interference effect in the older adults that was revealed in the response-locked analysis of the lemma retrieval task. Electrodes included in the cluster are marked in red. **Right**: Waveforms of the grand averages for the 1st (in blue) and 5th ordinal position (in red) for electrode CP4 in the older adults.

In the older adults, testing for a cumulative semantic interference effect in the latency range from 540 to 450 ms before response onset, the cluster-based permutation test revealed a difference between the first and fifth ordinal positions (*p* = 0.006) that was taken as evidence for the stage of lemma retrieval. The difference was most pronounced over left posterior electrodes during the first 60 ms and most pronounced over the right posterior electrodes during the last 50 ms of the effect. No effect was found in the stimulus-locked analysis. The scalp distribution and the waveforms of the first and fifth ordinal position’s grand average are shown in Figure 6.

**FIGURE 7:**
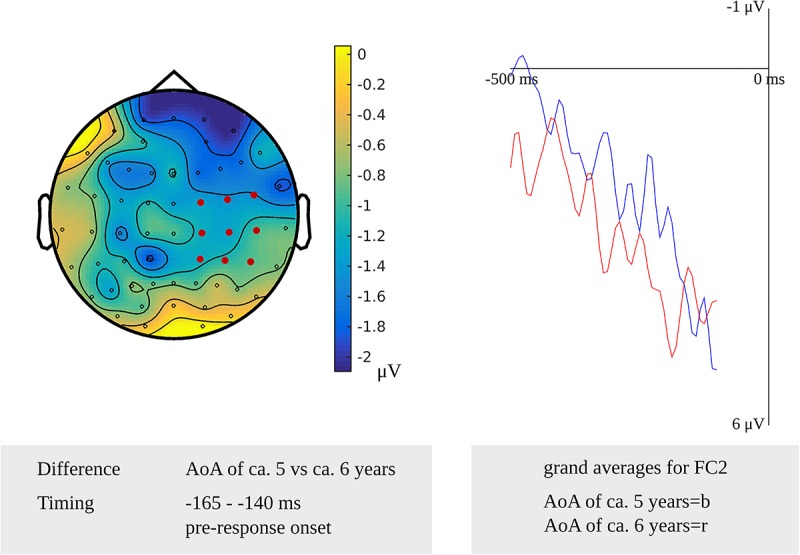
**Left**: The cluster related to the AoA effect in the older adults that was revealed in the response-locked analysis of the lexeme retrieval task. Electrodes included in the cluster are marked in red. **Right**: Waveforms of the grand averages for an AoA of ca. 5 (in blue) and 6 years (in red) for electrode FC2 in the older adults.

For lexeme retrieval, an AoA effect was revealed in the cluster-based permutation analysis in three response-locked time windows as a difference between items with an early AoA (of around 5 years) and items with a moderate AoA (of around 6 years). The AoA effect was most pronounced over centro-posterior electrodes in the earliest cluster from 430 to 420 ms (*p* = 0.012) before response onset. In the second cluster, from 210 to 195 ms (*p* = 0.009) before response onset, the effect was most evident over the right frontal electrodes. The AoA effect was most distinct over right central electrodes in the last cluster with the longest duration from 165 to 140 ms (*p* = 0.013) before response onset, which is depicted in Figure 7. In Figure 7, the waveforms of the grand averages for the early and moderate AoA items are provided as well. No differences were found between items with an early AoA and items with a late AoA (of around 7 years). Also, no AoA effect was found in the stimulus-locked analysis.

**FIGURE 8:**
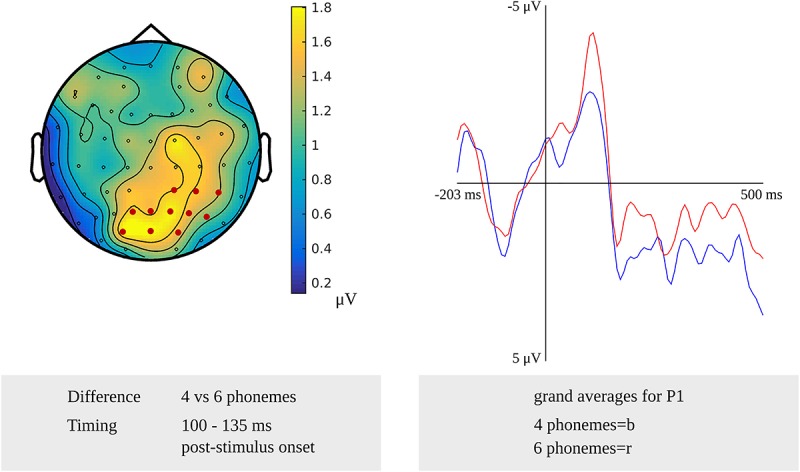
**Left**: The cluster related to the effect of non-word length in phonemes in the older adults that was revealed in the stimulus-locked analysis of the task targeting phonological encoding. Electrodes included in the cluster are marked in red. **Right**: Waveforms of the grand averages for a non-word length of four (in blue) and six phonemes (in red) for electrode P1 in the older adults.

For phonological encoding, the effect of the length in the number of phonemes on non-word reading was used in the cluster-based permutation analysis. In the older adults, a length effect was revealed as a difference between non-words with a length of four and six phonemes in the time windows from 100 to 135 ms (*p* = 0.019) and from 280 to 300 ms (*p* = 0.0038) after stimulus onset. In the first time window, the length effect was most pronounced over the right posterior electrodes, as shown in Figure 8. The waveforms of the grand averages for items consisting of four and six phonemes are provided in Figure 8 as well. The effect was most pronounced over bilateral frontal and central electrodes in the second time window. No effects were found for the comparison of non-words with a length of four and five phonemes. Also, no length effects were found in the response-locked analysis.

**FIGURE 9:**
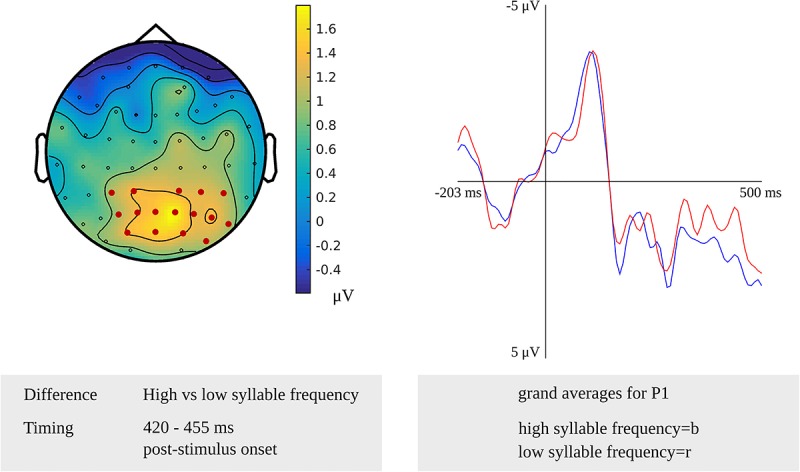
**Left**: The cluster related to the syllable frequency effect in the older adults that was revealed in the stimulus-locked analysis of the task targeting phonetic encoding. Electrodes included in the cluster are marked in red. **Right**: Waveforms of the grand averages for a high (in blue) and low syllable frequency (in red) for electrode P1 in the older adults.

For tapping into phonetic encoding, the effect of syllable frequency on the non-word reading task was used. The stimulus-locked cluster-based permutation analysis revealed a syllable frequency effect for reading non-words with a high syllable frequency (ranging from 1,000 to 1,500) as compared to reading non-words with a moderate syllable frequency (ranging from 500 to 1,000) in a time window from 280 to 300 ms (*p* = 0.0094) and in a time window from 365 to 375 ms (*p* = 0.022) after stimulus presentation. The earliest effect was most pronounced over electrodes covering the right hemisphere, the later effect over the posterior electrodes. Furthermore, the comparison of non-words with a high syllable frequency to non-words with a low syllable frequency (ranging from 250 to 500) revealed effects from 280 to 290 ms (*p* = 0.0196) and from 420 to 455 ms (*p* = 0.0078) after stimulus onset. The effect starting at 280 ms was most pronounced over right-posterior electrodes, while the later effect shown in Figure 9 was most pronounced over bilateral posterior electrodes. The waveforms of the high- and low-frequency items’ grand averages are shown in Figure 9 as well. Also, the syllable frequency effect was revealed from 455 to 435 ms (*p* = 0.016) before response onset. This effect was most pronounced over bilateral frontal and central electrodes.

#### Differences Between Younger and Older Adults

**FIGURE 10:**
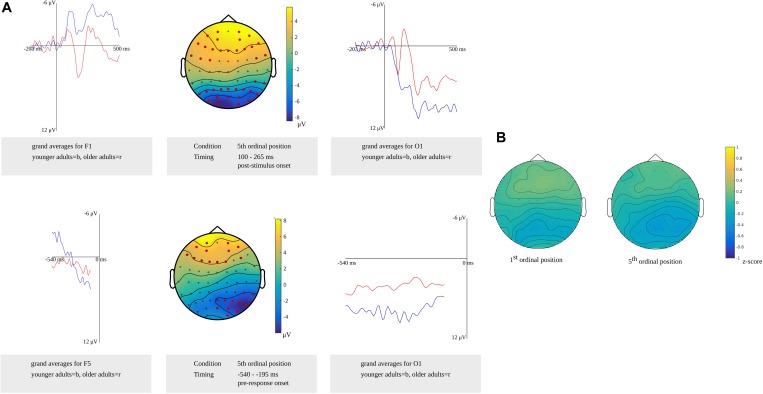
**(A)** Difference between younger and older adults identified in the stimulus-locked **(top)** and response-locked analysis **(bottom)** for the 5th ordinal position in the lemma retrieval task, showing a positive cluster over frontal electrode sites and a negative cluster over posterior electrode sites. Electrodes included in the clusters are marked in red. Waveforms of the grand averages for the younger (in blue) and older adults (in red) of the frontal electrodes F1 **(top left)** and F5 **(bottom left)** and posterior electrodes O1 **(right)**. **(B)** Scalp distributions per ordinal position showing the *z*-scores of the older adults compared to the younger adults.

Comparing the older and younger adults in the time window for lemma retrieval in younger adults from 100 to 265 ms after stimulus presentation in the fifth ordinal position, the cluster-based permutation analysis showed that both groups differed. In this time window, two effects were identified: a positive (*p* = 0.0026) and a negative one (*p* = 0.0022). The electrodes over which the positive effect was most pronounced were located in frontal regions bilaterally. The negative effect was most pronounced in bilateral posterior regions. Also, in the time window for lemma retrieval in older adults from 540 to 450 ms before response onset, both groups were found to differ. Differences were observed as a positive (*p* = 0.023) effect that was most pronounced over bilateral frontal electrodes and a negative effect (*p* = 0.013) that was most pronounced over bilateral posterior electrodes. Furthermore, a difference between the groups was observed in the response-locked time window for lemma retrieval in the younger adults from 445 to 195 ms before response onset (*p* = 0.0044). This difference was most pronounced in the posterior regions bilaterally. The clusters are shown in Figure 10A along with the waveforms of the grand averages for younger and older adults.

Based on the results from the cluster-based permutation analysis, a time window from 540 to 450 ms before response onset in older adults was compared to a time window from 365 to 275 ms before response onset in young adults. The *z*-scores computed for the first (*M* = 0.03, *SD* = 0.15, range = −0.37 to 0.27) and the fifth ordinal positions (*M* = −0.12; *SD* = 0.15, range = −0.41 to 0.19) indicated no differences in scalp distributions between the older and the younger adults. Figure 10B shows the *z*-scores of the individual electrodes mapped onto the scalp distribution per ordinal position.

**FIGURE 11:**
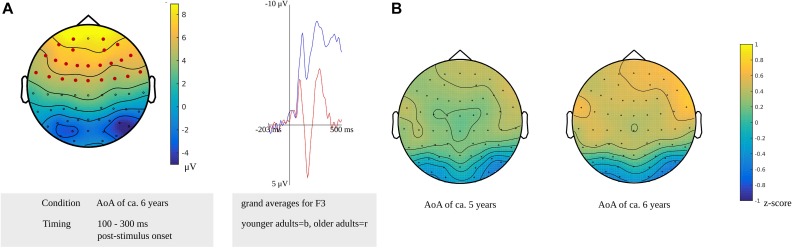
**(A)** Left: Cluster related to the difference between younger and older adults identified in the stimulus-locked analysis for an AoA of ca. 6 years in the lexeme retrieval task. Electrodes included in the cluster are marked in red. Right: Waveforms of the grand averages for the younger (in blue) and older adults (in red) of the electrodes F3. **(B)** Scalp distributions per AoA showing the *z*-scores of the older adults compared to the younger adults.

In the time window for lexeme retrieval identified for the younger adults, from 100 to 300 ms after stimulus presentation, a difference between the older and younger adults was found for items with a moderate AoA (*p* = 0.0022). The difference was most pronounced in frontocentral regions bilaterally, as shown in Figure 11A. Also, the waveforms of the younger and older adults’ grand averages are provided in Figure 11A. The response-locked time windows for lexeme retrieval from 430 and 140 ms before response onset identified in the older adults and from 475 to 330 ms before response onset identified in the younger adults did not reveal any differences between the groups.

The cluster-based permutation analysis targeting lexeme retrieval revealed no difference between early and late AoA conditions in the older adults; thus, the scalp distributions of the age groups could not be compared on these conditions. The age groups were compared on the early AoA and the moderate AoA conditions. A time window from 175 to 225 ms after stimulus presentation in the younger adults was compared to a time windows from 430 to 420 ms, from 210 to 195 ms, and from 165 to 140 ms before response onset in the older adults. Based on the *z*-scores of the electrodes, no differences in scalp distributions were found between the older and the younger adults for the early AoA (*M* = 0.15, *SD* = 0.26, range = −0.64 to 0.64) and the moderate AoA conditions (*M* = 0.29, *SD* = 0.33, range = −0.64 to 0.89). This is shown in Figure 11B.

**FIGURE 12:**
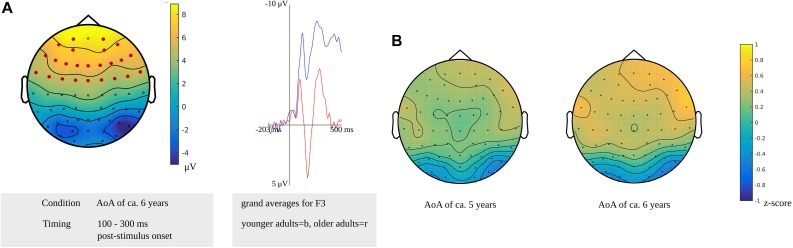
**(A)** Left: Clusters related to the difference between younger and older adults identified in the stimulus-locked analysis for a non-word length of five (top) and six (bottom) phonemes in the task targeting phonological encoding. Electrodes included in the clusters are marked in red. Right: Waveforms of the grand averages for the younger (in blue) and older adults (in red) for the electrodes P4. **(B)** Scalp distributions per non-word length in phonemes showing the *z*-scores of the older adults compared to the younger adults.

The cluster-based permutation analysis for phonological encoding showed differences between older and younger adults for non-words consisting of five phonemes in a time window from 350 to 415 ms after stimulus presentation (*p* = 0.015). Also, for the non-words consisting of six phonemes, a difference between both age groups was found from 390 to 425 ms after stimulus presentation (*p* = 0.014). Both time windows were identified for phonological encoding in the young adults. The differences were most pronounced in bilateral posterior regions, as shown in Figure 12A. Figure 12A also shows the waveforms of the grand averages of the younger and the older adults. In the time windows identified for the older adults, no differences between the groups were found. This result was also the case for the response-locked time windows identified for phonological encoding in the younger adults.

For the older adults, no difference was found between non-words composed of four and five phonemes in the cluster-based analysis targeting phonological encoding, so the age groups cannot be compared on these conditions. The conditions with four and six phonemes were included in the scalp distributions analysis. Time windows from 390 to 425 ms after stimulus presentation and from 330 to 320 ms before response onset in the younger adults were compared to time windows from 105 to 135 ms and from 280 to 295 ms after stimulus presentation in the older adults. The *z*-scores revealed no differences in scalp distributions between the older and the younger adults for the four phonemes condition (*M* = −0.24, *SD* = 0.20, range = −0.74 to 0.12) and the six phonemes condition (*M* = −0.21, *SD* = 0.20, range = −0.74 to 0.11). The scalp distributions are shown in Figure 12B.

**FIGURE 13:**
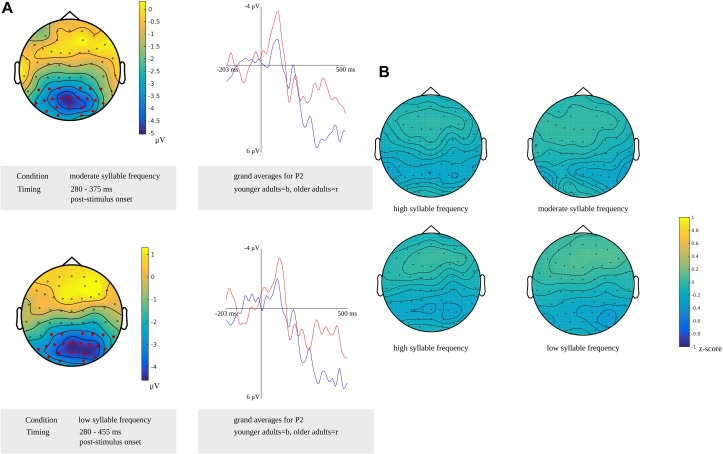
**(A)** Left: Clusters related to the difference between younger and older adults identified in the stimulus-locked analysis for a moderate (top) and high syllable frequency (bottom) in the reading task targeting phonetic encoding. Electrodes included in the clusters are marked in red. Right: Waveforms of the grand averages for the younger (in blue) and older adults (in red) for the electrodes P2. **(B)** Scalp distributions for high and moderate syllable frequency (top) and for high and low syllable frequency (bottom) showing the *z*-scores of the older adults compared to the younger adults.

For phonetic encoding, the cluster-based permutation analyses showed a difference between the older and the younger adults for moderate frequency non-words from 280 to 375 ms after stimulus presentation (*p* = 0.007). This range corresponds to the time window identified for phonetic encoding in the older adults. The groups did not differ in the time window for the younger adults. For low-frequency non-words, a difference between both groups was found from 280 to 455 ms after stimulus presentation (*p* = 0.011). This time window corresponds to the time window identified for phonetic encoding in older adults and also includes the time window in which phonetic encoding was identified in younger adults. Both effects were most pronounced in bilateral posterior regions, as shown in Figure 13A. This figure also shows the waveforms of the grand averages for the younger and older adults. No differences between the groups were found in the response-locked time windows.

For non-words with a high syllable frequency and a moderate syllable frequency, a time window from 410 to 440 ms after stimulus presentation in younger adults was compared to time windows from 280 to 300 ms and from 365 to 375 ms after stimulus presentation in older adults. Based on the *z*-scores, no differences in scalp distributions were found between the older and the younger adults for both high frequency (*M* = −0.15, *SD* = 0.11, range = −0.33 to 0.10) and moderate frequency conditions (*M* = −0.11, *SD* = 0.11, range = −0.36 to 0.12). Also, *z*-scores for non-words with a high syllable frequency and a low syllable frequency were computed to compare a time window from 385 to 440 ms after stimulus presentation in younger adults to time windows from 280 to 290 ms and from 420 to 455 ms after stimulus presentation and from 450 to 460 ms before response onset in older adults. For the high-frequency (*M* = −0.15, *SD* = 0.12, range = −0.36 to 0.18) and the low-frequency conditions (*M* = −0.11, *SD* = 0.14, range = −0.44 to 0.17), no differences in scalp distributions based on the *z*-scores were found between older and younger adults. The scalp distributions are shown in Figure 13B.

## Discussion

The current study had two aims, which will be addressed in this discussion. The first was to identify the speech production stages in a group of older adults and in a group of younger adults. The second aim was to test whether the stages change with age with respect to the timing or regarding the neural configuration observed in the scalp distributions.

### Identification of Speech Production Stages

To identify the stages of the speech production process, a protocol with EEG was developed with three tasks tapping into four speech production stages. The manipulations in the tasks used to identify the stages had an effect on the response times in both the older and the younger adults. In the lemma retrieval task, the cumulative semantic interference effect caused increased response times for items belonging to the same category when they were presented at the fifth ordinal position compared to when they were presented at the first ordinal position. Also, later response times were found for items with a later AoA compared to items with an earlier AoA, as shown in the lexeme retrieval task. In the non-word reading task, non-words that consisted of more phonemes used to track phonological encoding and non-words with a lower syllable frequency used to tap into phonetic encoding caused increased response times. The results of the cluster-based permutation analysis of the EEG data revealed that the manipulations used in the tasks of the protocol showed an effect in particular time windows. First, the time windows in the younger adults will be discussed, after which the time windows in the older adults will be addressed.

#### Younger Adults

In the younger adults, the timing of the cumulative semantic interference effect was revealed from 100 to 265 ms after stimulus presentation and from 445 to 195 ms before response onset. Response-locked cumulative semantic interference effects have not been reported in previous studies using EEG. However, the stimulus-locked timing largely corresponded to the timing of this effect found by Maess et al. (2002) from 150 to 225 ms after stimulus presentation, but only partially overlapped with the timing of this effect found by Costa et al. (2009) from 200 to 380 ms after stimulus presentation. As our materials showed, the items used by Maess et al. (2002) depicted mono- and disyllabic high-frequency words. The materials used by Costa et al. (2009) also included longer and less-frequent words, which may explain the later latency of the cumulative semantic interference effect.

The timing of the AoA effect for the younger adults appeared from 100 to 300 ms after stimulus presentation. This result corresponds to the timing of this effect from 120 to 350 ms after stimulus presentation found by Laganaro and Perret (2011). Also, the response-locked effect for the younger adults from 475 to 330 ms before response onset overlaps with previously reported time windows of this stage from 380 after stimulus presentation up to 200 ms (Laganaro et al., 2012) or up to 100 ms before response onset (Valente et al., 2014).

Non-word length in phonemes was found to have an effect from 350 to 425 after stimulus presentation and from 335 to 320 before response onset for the younger adults. No previous speech production studies using EEG have reported on non-word length effects. Word length effects have been studied using picture-naming tasks, but no effects have been identified (Valente et al., 2014; Hendrix et al., 2017). In our study, a length effect was identified with a non-word reading task. The input for phonological encoding of a word differs from the input for phonological encoding of a non-word, which may explain why the effect was found for non-words, but not for words. The phonological encoding of a familiar lexeme likely required less effort than the phonological encoding of an unfamiliar string of phonemes.

The syllable frequency effect in the non-word reading task has been identified after stimulus presentation from 350 to 450 ms for younger adults. Also, the effect has been found before response onset from 250 to 200 ms. Bürki et al. (2015), using syllable frequency effect in a non-word reading task, identified this effect from 170 to 100 ms before response onset. This effect was later than the effect found in the current study, most likely because the task required participants to insert a phoneme into the non-word as they read it, which complicated the task.

The time windows described in the previous paragraphs correspond to the speech production stages identified by Levelt et al. (1999) and Indefrey (2011). In the speech production model, lemma retrieval precedes lexeme retrieval. In the younger adults, the cumulative semantic interference effect and the AoA effect started at the same time in the stimulus-locked analysis, but the AoA effect lasted longer than the cumulative semantic interference effect. In the response-locked analysis, the cumulative semantic interference effect lasted longer than the AoA effect. The time window for lexeme retrieval started before and ended during the time window for lemma retrieval. In the lexeme retrieval task, lemma retrieval was not manipulated, and thus, lemma retrieval was less demanding (and, hence, faster) in the lexeme retrieval task than in the lemma retrieval task. Therefore, the time window for lexeme retrieval in the lexeme retrieval task may have started earlier than the time window for lemma retrieval in the lemma retrieval task.

Lexeme retrieval is followed by phonological encoding in the model. For picture naming, the lexical route is used, whereas for non-word reading, the sublexical route should be recruited. Thus, the timing of the lexeme retrieval stage in the picture-naming task and the timing of the phonological encoding stage in the non-word reading task cannot be compared using our method. Phonological encoding precedes phonetic encoding in the model. In the stimulus-locked analysis, the non-word length effect started at the same time as the syllable frequency effect, but the length effect ended earlier. In the response-locked analysis, the non-word length in phonemes effect preceded the syllable frequency effect. Thus, the protocol can be used to identify the stages using EEG in the younger adults.

#### Older Adults

In the older adults, the cumulative semantic interference effect was found from 540 to 450 ms before response onset. Since no response-locked cumulative semantic interference effects have been reported previously, the response-locked effect revealed in the older adults cannot be compared to other studies.

AoA effects have previously been identified in response-locked time windows until 200 ms (Laganaro et al., 2012) or 100 ms before response onset (Valente et al., 2014). These time windows overlap with the response-locked effects for the older adults from 430 to 140 ms before response onset.

The effect of non-word length in phonemes was identified from 100 to 135 ms and from 280 to 300 ms after stimulus presentation for the older adults. This study is the first to report the effects of non-word length in number of phonemes in an EEG study.

The second effect that was tested in the non-word reading task was syllable frequency, which has been identified from 280 to 455 ms after stimulus presentation. This effect was found from 455 to 435 ms before response onset as well. The timing of these effects is earlier than the timing of the syllable frequency effect reported by Bürki et al. (2015). As said above, task was more demanding, which may explain these differences.

In the older adults, the response-locked cumulative semantic interference effect preceded the response-locked AoA effect. This corresponds to the speech production processes identified by Levelt et al. (1999), Indefrey (2011), in which lemma retrieval precedes lexeme retrieval. In the older adults, the effect of non-word length in phonemes was identified before the syllable frequency effect, but there is an overlap of 20 ms in the stimulus-locked analysis. This finding is also in agreement with the model, because phonological encoding precedes phonetic encoding. Thus, the protocol can be used to identify the stages using EEG in the older adults as well.

### Aging Effects on Speech Production Stages

The behavioral data showed that both the younger adults and the older adults performed at ceiling on every task. Thus, in contrast to the study by Connor et al. (2004), no reduced accuracy in picture naming was found for older adults. This can be explained by a major difference in the age range of the participants in both studies: it was larger in the study by Connor et al. (2004: from 30 to 94 years) than in the current study, from 17 to 65 years. A behavioral difference between the groups was found in the response times. The older adults responded later than the younger adults on every task. It was hypothesized that the later response times of the older adults should reflected in the timing of the speech production stages in the EEG.

#### Differences in Timing Between Younger and Older Adults

Lemma retrieval requires semantic memory to activate the target lemma node along with its semantically related neighbors. These neighbors are inhibited to select the target lemma. Since both semantic memory (Cardenas et al., 2011; Harada et al., 2013) and inhibition (Harada et al., 2013) decline with aging, the duration of the lemma retrieval stage was expected to be increased in older adults. This hypothesis was not confirmed, because the lemma retrieval stage lasted 90 ms in the older adults, while in the younger adults, its duration was 165 ms in the stimulus-locked analysis and 250 ms in the response-locked analysis. However, all time windows of the effects that were found in the older adults were shorter than the time windows of the effects found in the younger adults. In older adults, neurons that fire together are possibly less synchronous in their timing, less aligned regarding their geometry, or the effect has a more variable latency (Wlotko et al., 2010). Therefore, the time window in which all participants show an effect is shorter.

Since the duration of lemma retrieval was expected to be increased, the onset of the next stage, lexeme retrieval, was expected to be delayed in the older adults. This hypothesis was confirmed. The response-locked effect started 45 ms later for the older adults compared to the younger adults. Also, an increased duration of the lexeme retrieval stage was hypothesized, because of the tip-of-the-tongue phenomenon, which is observed more frequently in older adults (Shafto et al., 2007). No increased duration was found, which again can be explained by the reduction in the effect caused by the effect’s variability within and between the older adults (Wlotko et al., 2010).

The stages of the sublexical route were expected not to be delayed in older adults. There have been no previous studies on aging’s effect on phonological encoding. Also, older adults have not revealed longer response times producing alternating syllable strings, which require more effort during phonetic encoding, than for the production of sequential syllable strings (Tremblay and Deschamps, 2016). However, both the effect of non-word length in phonemes related to phonological encoding and the syllable frequency effect targeting phonetic encoding started earlier for the older adults than for the younger adults. The difference in the onset of the timing of these stages between the groups is quite large; hence, this difference cannot be explained by the effect’s variability in older adults.

#### Neurophysiological Differences Between Younger and Older Adults

There were differences between the younger and the older adults regarding the time windows in which effects that were related to the stages were found. Results of the cluster-based permutation analyses showed that for every stage in at least one time window, differences between younger and older adults were found. In the time windows in which the younger adults showed a cumulative semantic interference effect, an AoA effect, or an effect of non-word length in number of phonemes, no such effect was observed in the older adults. This finding shows that the older adults had a different timing for the speech production stages than the younger adults. Despite partially overlapping time windows for the syllable frequency effect in the younger and older adults, a difference between both groups was found. The overlap in timing was possibly too short, so both groups differed during the majority of the time window, or the neural configuration of the syllable frequency effect differed between the groups. Except for the response-locked time windows identified using the cumulative semantic interference effect, differences between younger and older adults were generally identified in stimulus-locked time windows. When the stimulus is presented, the first process is the visual analysis of the picture or the non-word. This process is assumed to be identical in both age groups, because the efficiency of the visual network is not expected to change with age (Geerligs et al., 2015). After that, higher cognitive function networks, such as CON and FPCN are involved in the speech production stages. A decrease in the local efficiency of these networks may alter their neural signature or change their timing, which is reflected in the EEG. Even though the older participants in the study by Geerligs et al. were, on average, almost a decade older than the older adults in our study, our older participants may have a mild decrease in local efficiency and modularity in the CON and the FPCN compared to the younger adults, because the decrease is not linear with age (Geerligs et al., 2015).

**FIGURE 14:**
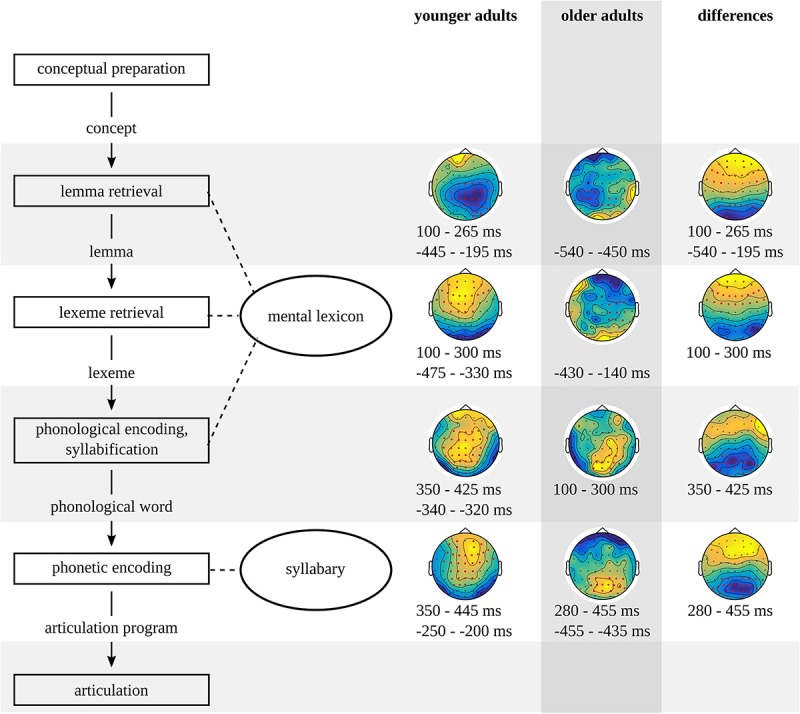
Timing of the stages in the model of spoken word and non-word production based on the results of the younger and the older adults and their differences.

An overview of the timing of the stages in the younger and older adults and the timing of significant differences between the two groups is provided in Figure 14.

Apart from the timing of the speech production stages, the neural configurations of the scalp distributions of the stages have been compared between the older and the younger adults. It was hypothesized that the scalp distributions do not change with age, because the same groups of neurons are expected to be involved in the stages of speech production in neurologically healthy adults, regardless of the adults’ age. Despite the fact that the effects related to each stage have been found in different time windows in the two groups, the scalp distributions during the stage were identical in the older and younger adults. This uniformity was the case for each speech production stage. Therefore, it can be concluded that older adults used the same neuronal processes as younger adults in the speech production stages. This was also supported by our behavioral results. Like the younger adults, the older adults performed at ceiling on the tasks. Also, the response times showed that the manipulations used in the tasks had the same effects in older and younger adults. Thus, the same factors had an influence on the speech production stages in both age groups.

The question remains why the response times of the older adults were later than the response times of the younger adults, even though the timing of the effects used to target the speech production stages was not generally delayed in the older adults. In the lexical route, lexeme retrieval was found to be delayed in older compared to younger adults. Since both picture-naming tasks required lexeme retrieval, the delay before this stage may have resulted in longer response times on the lemma and lexeme retrieval tasks. This is in line with the findings in the study by Laganaro et al. (2012) revealing differences between slow and fast speakers before the time window in which the AoA effect was found.

Lexeme retrieval is not involved in non-word production Therefore, delayed lexeme retrieval cannot explain later response times on non-word tasks in older adults, while no delay was observed for the phonological and phonetic encoding stages. Maybe, older adults respond later, because they generally are slower, as suggested in the Global Slowing Hypothesis (e.g., Brinley, 1965). However, this should have been reflected in the EEG as a longer duration and a later onset for every speech production stage, because neurophysiological measures are more sensitive than response time measures. Participants were asked to name the items as fast and accurately as possible. The tasks were fairly easy, so the accuracy of all patients was at ceiling. While younger adults can respond fast and accurately at the same time, older adults are known to focus on either speed or accuracy (Ratcliff et al., 2007). Maybe older adults focused more on accuracy in our study and, therefore, needed to collect more information before they were ready to respond (Rabbitt, 1979). In that case, the processes may not have been delayed in general, but only the decision whether the response was accurate or not was delayed. Thus, after the speech production process has been planned to its final stage, articulation, the older adults may have waited longer than the younger adults until they responded. In that case, this effect is not visible on the EEG, but only reflected in longer response times. If older adults wait before responding, the response-locked effects should be identified earlier in the older adults than in the younger adults. This, indeed, was the case for the cumulative semantic interference effect and the syllable frequency effect, but not for the AoA effect. However, individual differences are known to modulate the time window of the AoA effect (Laganaro et al., 2012). A possible modulation of the AoA effect is supported by our response time data, in which the older adults showed a smaller AoA effect than the younger adults.

## Conclusion

To conclude, the stages of the speech production process have been successfully identified in older and younger adults using the tasks of the protocol with EEG. The manipulations in the tasks had the same effect on the response time in both age groups; thus, the same factors influenced the speech production stages. Also, the scalp distributions related to the speech production stages did not differ between the older and the younger adults. This shows that the same neural processes are used during the speech production stages.

However, behaviorally, the comparison of the older and the younger adults showed that the older adults required longer response times on all tasks. Yet, the EEG results showed that the speech production stages do not generally start later or last longer in the older adults compared to the younger adults.

## Limitations

The study is subject to two potential limitations. In this study, we included older adults (40–65 years old), whereas it is common practice to compare younger adults (i.e., university students) to a group of elderly (usually over 70 years old). Thus, the age difference between the younger and older adults was smaller than in other studies that compare language production and, therefore, the aging effects found in the current study are potentially not as large as when younger and individuals with aphasia is now possible: individuals with aphasia and without concomitant cognitive disorders are usually within the age range of our group of older adults. However, it would be very interesting to compare the performance of both age groups of the current study with the healthy elderly and individuals with dementia, who are usually above 70 years old.

Second, non-word reading skills of the two groups included in the present study have not been assessed prior to the experiment. Reading was only assessed using self-report, which cannot be used to detect potential variation in reading skills. This potential variation may have had an effect at the phonological and phonetic encoding stages. We do not think this caveat influenced the results, however, because all participants performed at ceiling on the non-word reading task.

## Ethics Statement

This study was approved by the Research Ethics Committee of the Faculty of Arts of the University of Groningen.

## Author Contributions

JH is working on this Ph.D. project, did the actual studies, and wrote the largest part of the text. RB is promotor and PI of this project, and wrote a large part of the manuscript. RJ is daily supervisor of JH. PM initiated this project.

## Conflict of Interest Statement

The authors declare that the research was conducted in the absence of any commercial or financial relationships that could be construed as a potential conflict of interest.

## Footnotes

1 In fact, two non-word tasks were administered: reading and repetition. Since reading is more closely related to object naming (a visually presented stimulus evoking a spoken output), the data of the repetition task will be ignored.

## Appendix

**APPENDIX 1A:** EEG statistics for the younger adults.

**Analysis**	**Comparison**	**Time domain**	**Probability**	**Cluster statistics**	**Standard deviation**	**Confidence interval range**
**Lemma retrieval**						
Stimulus locked	1st vs. 5th ordinal position	100 to 265 ms	<0.001	−1,505.0	<0.001	<0.001
Response locked	1st vs. 5th ordinal position	−445 to −195 ms	0.005	−2,836.6	<0.001	0.002
**Lexeme retrieval**						
Stimulus locked	AoA ca. 5 years vs. ca. 6 years	100 to 300 ms	0.002	1,116.1	<0.001	0.001
Response locked	AoA ca. 5 years vs. ca. 7 years	−475 to −330 ms	<0.001	−1,954.7	<0.001	<0.001
**Phonological encoding in reading**						
Stimulus locked	Length 4 vs. 5 phonemes	350 to 415 ms	0.003	665.8	<0.001	0.002
	Length 4 vs. 6 phonemes	390 to 425 ms	0.005	317.9	<0.001	0.002
Response locked	Length 4 vs. 5 phonemes	−335 to −320 ms	0.008	200.7	0.001	0.002
	Length 4 vs. 6 phonemes	−330 to −320 ms	0.008	117.0	0.001	0.002
**Phonetic encoding in reading**						
Stimulus locked	High vs. moderate frequency	400 to 450 ms	0.020	316.5	0.002	0.004
	High vs. low frequency	350 to 450 ms	0.012	665.4	0.002	0.003
Response locked	High vs. low frequency	−250 to −200 ms	0.021	214.7	0.002	0.004

## Appendix

**APPENDIX 1B:** EEG statistics for the older adults.

**Analysis**	**Comparison**	**Time window**	**Probability**	**Cluster statistics**	**Standard deviation**	**Confidence interval range**
**Lemma retrieval**						
Response locked	1st vs. 5th ordinal position	−540 to −450 ms	0.006	−340.9	0.004	0.007
**Lexeme retrieval**						
Response locked	AoA ca. 5 vs. ca. 6 years	−430 to −420 ms	0.012	−78.8	0.002	0.003
		−210 to −195 ms	0.009	−96.7	0.001	0.003
		−165 to −140 ms	0.013	−131.6	0.002	0.003
**Phonological encoding in reading**						
Stimulus locked	Length 4 vs. 6 phonemes	100 to 135 ms	0.020	124.7	0.002	0.004
		280 to 300 ms	0.004	186.8	<0.001	0.002
**Phonetic encoding in reading**						
Stimulus locked	High vs. moderate frequency	280 to 300 ms	0.009	142.7	0.001	0.003
		365 to 375 ms	0.022	46.5	0.002	0.004
	High vs. low frequency	280 to 290 ms	0.020	59.2	0.002	0.004
		420 to 455 ms	0.008	174.2	0.001	0.002
Response locked	High vs. low frequency	−455 to −435 ms	0.016	98.6	0.002	0.004

## Appendix

**APPENDIX 1C:** EEG statistics for the comparison of the older and younger adults.

**Analysis**	**Condition**	**Time window**	**Probability**	**Cluster statistics**	**Standard deviation**	**Confidence interval range**
**Lemma retrieval**						
Stimulus locked	5th ordinal position	100 to 265 ms	0.003	907.6	<0.001	0.001
			0.002	−1,088.3	<0.001	0.001
Response locked	5th ordinal position	−540 to −450 ms	0.023	255.8	0.002	0.004
			0.013	−436.6	0.002	0.003
		−445 to −195 ms	0.004	−2,139.8	<0.001	0.002
**Lexeme retrieval**						
Stimulus locked	AoA ca. 6 years	100 to 300 ms	0.002	−1,749.4	<0.001	0.001
Phonological encoding in reading						
Stimulus locked	5 phonemes	350 to 415 ms	0.015	−386.5	0.002	0.003
	6 phonemes	390 to 425 ms	0.014	−227.5	0.002	0.003
**Phonetic encoding in reading**						
Stimulus locked	Moderate frequency	280 to 375 ms	0.007	−683.6	0.001	0.002
	Low frequency	280 to 455 ms	0.011	−904.9	0.002	0.003
